# The role of age, ethnicity and environmental factors in modulating malaria risk in Rajasthali, Bangladesh

**DOI:** 10.1186/1475-2875-10-367

**Published:** 2011-12-15

**Authors:** Ubydul Haque, Ricardo J Soares Magalhães, Dipak Mitra, Korine N Kolivras, Wolf-Peter Schmidt, Rashidul Haque, Gregory E Glass

**Affiliations:** 1 International Center for Diarrhoeal Disease Research Bangladesh, 68 Shaheed Tajuddin Ahmed Sharani, Mohakhali, Dhaka 1212, Bangladesh; 2 Department of Mathematical Sciences and Technology, Norwegian University of Life Sciences, Ås, Norway; 3 School of Population Health, University of Queensland, Herston, Queensland, Australia; 4 Department of Geography, Virginia Tech, Blacksburg, VA 24061, USA; 5 Environmental Health Group, Disease Control & Vector Biology Unit, London School of Hygiene and Tropical Medicine, London, UK; 6 Department of Molecular Microbiology and Immunology, John Hopkins Bloomberg School of Public Health, Baltimore, MD 21205, USA

## Abstract

**Background:**

Malaria is endemic in the Rajasthali region of the Chittagong Hill Tracts in Bangladesh and the Rajasthali region is the most endemic area of Bangladesh. Quantifying the role of environmental and socio-economic factors in the local spatial patterns of malaria endemicity can contribute to successful malaria control and elimination. This study aimed to investigate the role of environmental factors on malaria risk in Rajasthali and to quantify the geographical clustering in malaria risk unaccounted by these factors.

**Method:**

A total of 4,200 (78.9%; N = 5,322) households were targeted in Rajasthali in July, 2009, and 1,400 individuals were screened using a rapid diagnostic test (Falci-vax). These data were linked to environmental and socio-economic data in a geographical information system. To describe the association between environmental factors and malaria risk, a generalized linear mixed model approach was utilized. The study investigated the role of environmental factors on malaria risk by calculating their population-attributable fractions (PAF), and used residual semivariograms to quantify the geographical clustering in malaria risk unaccounted by these factors.

**Results:**

Overall malaria prevalence was 11.7%. Out of 5,322 households, 44.12% households were living in areas with malaria prevalence of ≥ 10%. The results from statistical analysis showed that age, ethnicity, proximity to forest, household density, and elevation were significantly and positively correlated with the malaria risk and PAF estimation. The highest PAF of malaria prevalence was 47.7% for third tertile (n = 467) of forest cover, 17.6% for second tertile (n = 467) of forest cover and 19.9% for household density >1,000.

**Conclusion:**

Targeting of malaria health interventions at small spatial scales in Bangladesh should consider the social and socio-economic risk factors identified as well as alternative methods for improving equity of access to interventions across whole communities.

## Background

Malaria eradication is the ultimate goal of the World Health Organization (WHO) [1]. Most malaria endemic countries are presently shifting their efforts from malaria control to eradication [2]. The WHO has targeted eight to ten countries for elimination of malaria by 2015 and afterwards in all other endemic countries [1]. Although the long-term goal is world-wide eradication, there remains some debate over the feasibility of elimination in Africa and eleven Asian malaria endemic countries [2-5]. Accurate maps of malaria incidence are important tools in malaria control as they can guide interventions and assess their effectiveness. Risk maps and geo-spatial data are increasingly used to support malaria elimination [6].

The Bangladesh national malaria survey in 2007 indicated that malaria was endemic in 13 of 64 administrative districts and that the crude prevalence is 4.0% [7,8]. In Bangladesh, prevalence is very low across large parts of the country except in Chittagong Hill Tracts (CHT), where risk is especially high among marginalized and hard-to-reach communities [6]. Moderate-to-high risk remains in well-defined areas across Bangladesh [6]. Active case detection with rapid diagnosis tests (RDT) conducted in 2007 revealed that the total malaria prevalence in the CHT was 11.7% with a majority of infections (>90%) caused by *Plasmodium falciparum *[7]. One particular sub-district, Rajasthali, had a prevalence of 36% and is considered the area most endemic to malaria in CHT [7]. In 2009, another prevalence study carried out in Rajasthali found 11.5% of participants positive with malaria infection despite 2 years of an extensive intervention and control program supported by the Global Fund [9]. Malaria is transmitted in a broad range of eco-epidemiological settings in Bangladesh due to high species diversity and the presence of species displaying different ecological behaviors [10]. Successful malaria control and elimination in Bangladesh can only be achieved if the country can target efficiently the endemic foci. Most of these hotspots are situated in CHT, along the borders of India and Myanmar where 90% of total cases occurs [6-8,11]. These areas are remote, forested and populated by ethnic minorities living a traditional rural life. The vectors present in this forested area are *Anopheles baimai *(dirus), *Anopheles philippinensis, Anopheles vagus *and *Anopheles minimus *[10]. Malaria risk is related to environmental factors affecting these vectors including altitude, forest, household density, cultivation practices, urbanization, and distance from water bodies [9,11-13], but the role of these factors to malaria risk has not been quantified.

At present controlling malaria in Bangladesh is based on both preventing infections and on prompt effective treatment of clinical cases. Through the National Malaria Strategic Plan, the malaria and parasitic disease control unit supports malaria prevention and treatment services in 13 endemic districts of Bangladesh.

Predicting the abundance, as well as understanding the risk factors, spatial distribution, and spread of malaria in endemic settings can significantly contribute to local malaria control strategies. A deeper understanding of the role of landscape attributes in the spatial distribution of malaria is crucial so that appropriate local elimination efforts can be developed.

Malaria prevalence in Bangladesh shows a pronounced heterogeneity of transmission [6]. Models predict that unrecognized heterogeneity reduces the efficacy of disease control strategies [14]. While escalating control measures in disease hotspots may be very effective at reducing overall transmission. Recent efforts to develop pragmatic global maps, such as Malaria Atlas Project (MAP), suggests a new era of using maps to define regional populations at risk of malaria, that will guide future global malaria control and the distribution of funds [15]. The most modifiable cause of malaria risk is having two or fewer bed nets; non-modifiable risk factors are associated with sex, age, forest cover, elevation and household density [9]. The relative importance of a risk factor can be estimated using the PAF. The odds ratio and the relative risk can only measure the level of risk associated with the exposure to a risk factor. They never reflect the impact of the factor in a population. PAF is the proportional reduction in population disease that would occur if exposure to a risk factor were reduced over a specified time interval from the population. Many diseases are caused by multiple risk factors, and individual risk factors may interact in their impact on overall risk of disease. PAF estimates can provide some insight for policymakers in planning public health interventions. The goal of this study was to estimate the PAF for different environmental factors for malaria in Rajasthali that will generate key information as a new phase of malaria control in Bangladesh begins with the support of the Global Fund.

## Methods

### Study area

Covering a surface area of 145.04 km^2^, Rajasthali lies between 22°20' to 22°26' north latitude and 92°08' to 92°22' east longitude in CHT. It is a hilly, remote area covered with dense forest. The total population of Rajasthali is 24,097 with 5,322 households. Most households belong to ethnic clans leading a traditional rural life. All households (n = 5,322) in Rajasthali were georeferenced [16].

### Malaria prevalence, socio-economic and environmental data

The malaria prevalence data used in this analysis was collected during a 2009 malaria-prevalence survey in Rajasthali using a two-stage cluster sampling technique. A questionnaire survey was conducted at the same time to obtain demographic information, bed net usage and socio economic status. A full description of the survey has been published [9]. Blood samples from 1,400 (> 0-102 years of age) individuals were tested using rapid diagnosis test. Out of 1,400 samples, 161 (11.5%) were positive for either *P. falciparum *(158, 11.3%) or *Plasmodium vivax *(11, 0.8%) [9]. Geometrically corrected, cloud free Landsat 5 Thematic Mapper image was taken on January 23rd, 2010 (Path 136, Row 44) from the United States Geological Survey. Based on the review of existing literature and considering the importance of land cover types, a supervised classification was performed. The study area was classified into six categories (deep water, shallow water, brown open land, bright open land, forest, and grassland/bush) based on the maximum likelihood method for PG-Steamer (Pixoneer Geomatics Inc., Tae-jon). Ground-truth sites and known land-cover were identified from high-resolution Google Earth images. The proportion of forested area within 2 km from each of 1,400 sampled households was calculated using Arc GIS 9.3. Later the proportion of forested area was categorized into tertiles for the analysis. A Shuttle Rader Topographic Mission Digital Elevation Model (SRTM DEM) of 3 arc-second (approximately 90 m) resolution was used to estimate the altitude of each sampled household using ArcGIS 9.3. Local house density was also used for analysis. Data preparation and collation of these items has been described in detail [9].

### Ethical consideration

This study was reviewed and approved by both the research review committee and ethical review committee of International Center for Diarrhoeal Diseases Research Bangladesh (ICDDR, B). Written consent was taken from study participants or their caretakers [9].

### Data analysis

#### Assessing variables

All statistical analyses were carried out using the statistical software STATA 11 (Stata Corp., College Station, TX). Data included individual-level variables (Age, sex, tribe); household level variables (Household head education and occupation, number of bed nets, long lasting insecticidal net (LLIN) use, bed net treatment and household floor materials) and environmental variables (forest, altitude, household density). The statistical analysis was carried out in two phases using malaria infection status (based on rapid diagnostic test result) as the outcome variable of interest. Firstly, all individual, household and environmental variables were screened using univariate logistic regression for statistical association with malaria infection status based on a liberal p-value of 0.20 in the likelihood-ratio test. Secondly, all variables significant in the screening phase were considered for inclusion through a manual backward stepwise variable selection process in a multivariable logistic regression analysis. The criterion for removal of risk factors was based on statistical considerations using the likelihood ratio test with a significance level of *p *> 0.05. The variables age, sex, ethnicity, number of bed net, forest, altitude, household density and floor were retained for the final multivariate model

#### Assessing spatial autocorrelation

The semivariogram was used as a graphical representation of the spatial dependence in the data. The residuals of the final model (age, forest, altitude and household density) were extracted and screened for spatial dependence using semivariograms developed with the package geoR of the statistical software R version 2.9.0 (The R foundation for Statistical Computing, Vienna). The residuals of a model was also tested without the non-significant (*p *> 0.1) variables (sex, tribes, number of bed net, LLIN, bed net treatment, floor, household head education and occupation), to observe changes to the semivariograms.

#### Estimation of the population attributable fractions of malaria

The PAF of risk factors included in the final multivariable model was estimated using the AFLOGIT procedure of STATA [17]. PAF estimates the proportional amount of disease risk if all the risk factors were simultaneously eliminated from the population. The adjusted PAF highlights how much of the disease risk is attributed to all the factors included in the model. From the initial descriptive analysis, reference levels for each factor with the lowest risk were determined. That ensured that the PAFs were derived from positive associations with the outcome. A univariate analysis for each risk factor was performed. Then adjusted ORs, PAF and 95% CIs from multivariate models including all the statistically significant risk factors were reported.

## Results

**Figure 1:**
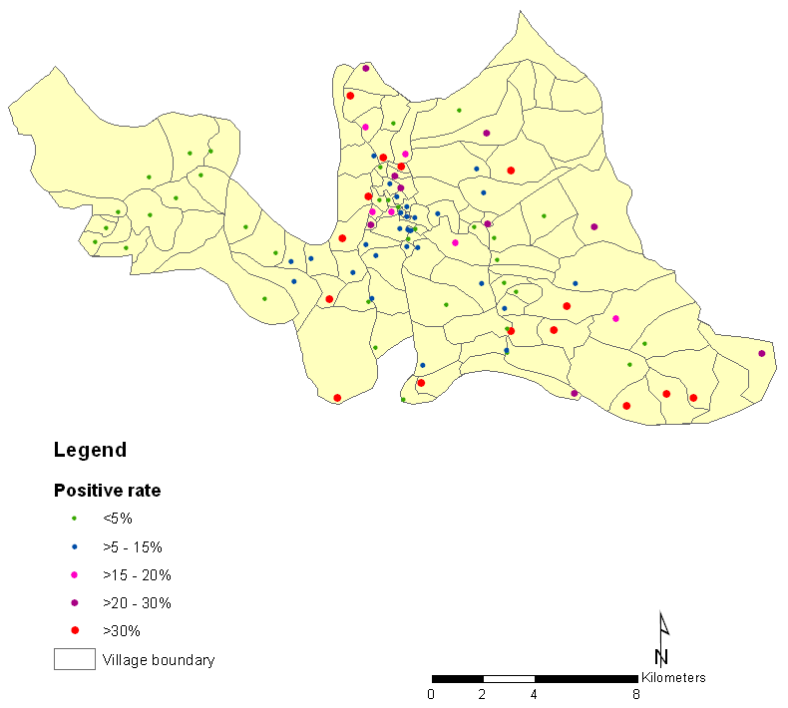
**Spatial distribution of malaria prevalence in Rajasthali region, Bangladesh for 2009**.

The spatial distribution of malaria prevalence in the 1,400 locations surveyed in Rajasthali shows that the distribution of malaria prevalence is heterogeneous across communities (Figure 1) ranging from 4.26% to 45.24% (Table 1). Out of 5322 households, 44.12% were in areas with malaria prevalence of ≥ 10%.

**Table 1:** Malaria prevalence distribution of households included in the 2009 malaria survey in the Rajasthali region, Bangladesh

Malaria prevalence	Number of households
4.26 - 8.92%	2912

> 8.92 - 14.22%	2213

> 14.22 - 20.65%	96

> 20.65 - 29.01%	79

> 29.01 - 45.24%	23

Age, sex, ethnic association, number of bed nets, forest, altitude, household density and floor type proved statistically significant in univariate analyses (Table 2). Only age, altitude, forest and household density proved statistically significant in the multivariate model. Children aged up to 10 years and people living in higher altitude areas had much higher odds of malaria infection than their peers. The odds of malaria increased with increasing elevation. People living around the densest forest (upper third tertile) were at a significantly higher risk of being infected compared to the first and second tertiles. In addition to age, altitude and forest, the odds of malaria also increased with increasing household density. In univariate analysis, lower household density (1-200) proved as the highest risk factor for infection. However, in the multivariate model, higher household density (>1000) produced an increased risk. Adjusted malaria risk with >1,000 households was significantly higher than for (1-200) households density (OR, 3.61 [95% CI, 1.34-9.74]; *P *= 0.011).

**Table 2:** Results of unadjusted and adjusted logistic regression model for RDT positive status

Covariates	Unadjusted OR (95% CI)	*P *value	Adjusted OR* (95% CI)	*P *value
**Ethnicity**				

Tripura	1.00		1.00	

Bengali	3.55 (1.48-8.48)	0.004	2.19 (0.87-5.52)	0.096

Marma	3.36 (1.43-7.87)	0.005	2.30 (0.94-5.59)	0.066

Other	2.56 (1.04-6.29)	0.040	1.72 (0.67-4.43)	0.258

**Altitude (meter)**				

≤ 50	1.00		1.00	

51 - 100	1.57 (1.10-2.25)	0.013	1.54 (0.94-2.51)	0.084

> 100	5.27 (3.06-9.08)	0.000	3.82 (1.88-7.76)	0.000

**Household density**				

1 - 200	1.00		1.00	

201 - 500	0.46 (0.29-0.71)	0.001	1.57 (0.83-2.97)	0.167

501 - 1000	0.40 (0.20-0.78)	0.007	3.12 (1.14-8.57)	0.027

> 1000	0.37 (0.25-0.55)	0.000	3.61 (1.34-9.74)	0.011

**Forest density**				

1^st ^Tertile (n = 466)	1.00		1.00	

2^nd ^Tertile (n = 467)	2.21 (1.32-3.70)	0.003	2.66 (1.52-4.66)	0.001

3^rd ^Tertile (n = 467)	4.62 (2.86-7.45)	0.000	9.02 (3.74-21.74)	0.000

**Age**				

>10 years	1.00		1.0	

0-10 years	2.75 (1.94-3.89)	0.000	2.60 (1.80-3.75)	0.000

*All variables included in the model

**Figure 2:**
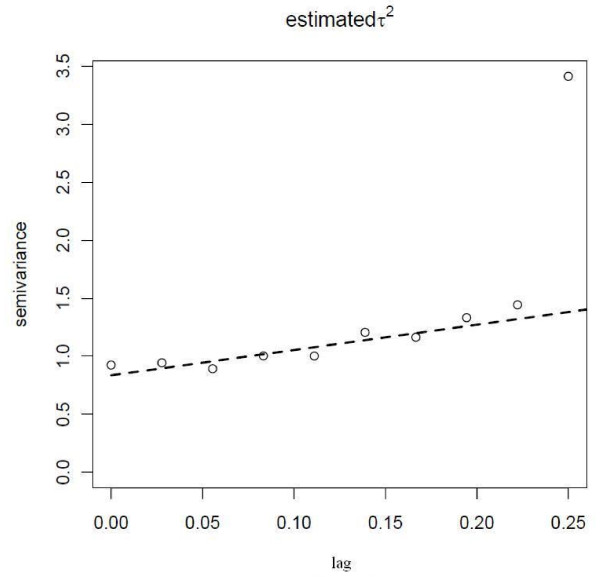
**Residual semivariogram of malaria prevalence in Rajasthali using the final multivariable model**.

After taking into account the effect of the individual- and survey-level environmental variables, there was little evidence for spatial dependence (Figure 2). For that reason, extra spatial parameters were not fitted to the regression.

### Population attributable fraction

The PAFs were estimated for the environmental covariates; altitude, population density and forest (Table 3). Adjusting for all other risk factors, the factors with the highest PAFs were the third tertile of forest cover (47.7%), being a member of the Marma community (23.8%) and living in a place where house density was > 1,000/sq km (19.9%). Most of the Marma villages are located in the western and central regions of Rajasthali. Household density is highest in the central area of Rajasthali where most of the administrative buildings are located. Forest density is higher in the eastern parts of Rajasthali. The second tertile of forest cover accounted for 17.6% of the PAF, Bengali people accounted for 14.5% and household elevation (altitude 51-100 m from sea level) accounted for 11.2% (Table 3). Other communities including Khiang and Chakma (7.3%) [16], and living at > 100 m altitude (9.3%) with lower household density (201-500) accounted for the 6.5% PAFs (Table 3).

**Table 3:** Population Attributable Fraction of positive rapid test result for malaria associated with environmental risk factors

Covariates	Population Attributable fraction	95% CI
**Tribe**		

Tripura (reference)		

Bengali	14.5%	-0.6% - 27.3%

Marma	23.8%	1.1% -41.2%

Other	7.3%	-4.6% - 17.8%

**Altitude (meter)**		

≤ 50 (Reference)		

51 - 100	11.2%	-1.9% - 22.6%

> 100	9.3%	3.4% - 14.3%

**Household density**		

1 - 200 (Reference)		

201 - 500	6.5%	-2.7% - 14.9%

501 - 1000	4.4%	0.2% - 8.3%

> 1000	19.9%	8.6% -29.9%

**Forest density**		

1^st ^Tertile (n = 466) (Reference)		

2^nd ^Tertile (n = 467)	17.6%	7.8% - 26.3%

3^rd ^Tertile (n = 467)	47.7%	36.1% - 57.2%

*estimated using the adjusted odds ratio

## Discussion

The purpose of this study was to estimate the PAFs for different contributors to malaria risk and to develop a local malaria risk model in a highly endemic area of Bangladesh. The results show that at local spatial scales there was little evidence to include environmental covariates (such as temperature and precipitation) in the models and only the covariates age, forest, altitude and household density were significantly associated with infection. Furthermore, there was little evidence for spatial dependence in semivariograms after the model was developed, suggesting little evidence of substantial unexplained variation that varied predictably over the region. Taken together these results suggest that locally, individual-level factors (e.g. socioeconomic factors; behavioral factors; adherence to preventative measures) are more likely to determine the spatial distribution of malaria infections. The dataset did not include additional individual-level variables describing behavior and further surveys aimed at developing spatial risk models at small spatial scales should endeavor to include this type of information. The inclusion of these individual-level variables may improve the discriminatory ability of the model but unfortunately were not available in the Rajasthali dataset. The greatest PAFs were altitude, household density and forest cover.

Presumably due to lower acquired immunity, children are more vulnerable for malaria infection [7,18-20]. Results from this study are consistent with the age related effects. The high PAFs for forest coverage and high altitude suggest a critical role of specific malaria vectors. In this region *An. dirus, An. minimus*, and a diverse fauna of other anopheline species have been reported as main malaria vectors, with *An. dirus spp *being an efficient vector in forests habitats [21,22]. Although the vector species have not been sampled in the study area, the higher risk of infection in hilly and forested area may implicate the primary vector species as *An. dirus*. Despite the small overall elevation distribution in Rajasthali (from 22 m to 359 m above the sea level), this factor may have some contribution to characterize differences of transmission risk, and malaria prevalence rate. Altitude proved one of the key factors responsible for malaria transmission in CHT [8]. Few studies have confirmed that elevation is one of the key factors associated with malaria [23-25]. Study results from Afghanistan showed no transmission in villages at elevations >2,000 m [26]. The altitude in all households of Rajasthali is < 360 m and, thus, not high enough to provide cooler temperatures.

Univariate analyses and PAF further revealed that lower household density was a significant risk factor for malaria infection. This pattern is similar to a study from Ghana that reported higher malaria risk in smaller villages and in outer areas of each village [27]. However, for the multivariate model, the effect of household density was reversed and definitive conclusions could not easily be made. Strong correlations were observed between the forest coverage and house density, and may account for the results in the multivariate model.

The relative importance of risk factors for public health intervention can be estimated using PAF. PAF defines the reduction of incidence and can be achieved if the population had been entirely unexposed compared to its current exposure pattern [28]. The results indicate that altitude, household density and forest were the most important risk factors associated with malaria prevalence in the study area.

These findings are important for targeted intervention and resource allocation. The greatest use of PAFs here has highlighted modifiable risk factors, predicting how much disease can be avoided with their elimination. The non-modifiable factors cannot be eliminated here (e.g., ethnicity). Modifiable risk factors should be considered to prioritize and target public health intervention strategies in CHT. The different ethnic community living in the very remote regions (close to deep forest and in high altitude areas) accounted for the highest PAFs. These results suggest that if living conditions and access to treatment care services can improve, then it would be possible to prevent a significant proportion of malaria incidence in this population. PAF estimates obtained in this study will be useful since the malaria control programme in Bangladesh is enhancing its control efforts and has committed to reduce malaria cases by 60% by 2015 as the baseline year/cases of 2015 [29].

The increasing rate of malarial infection with greater distances from the village centre could also be due to proximity to mosquito breeding sites. Residences on the village periphery may be located closer to swamps and agricultural fields that result in a greater local mosquito density, although we have no data to indicate this occurred. Socioeconomic status can be protective against malaria if it provides better access to anti-malarial medicines or bed nets, which is the ultimate priority in accordance to malaria control program in Bangladesh. In the central and western areas, it is possible that the more established families with more resources live near the town centre or market [27]. These trends could also be explained by the proximity of health clinics and markets that sell bed nets and anti-malarial medications to the village centre. These findings are pivotal for national control programs and could help policy makers identify high-risk areas that can be targeted first (Figure 1). The calculations (Table 3) are then able to inform program managers if they need to re-distribute or recruit additional health workers to cover 100% of the population in this area. It has important implications in meeting the first key objective of the control programme, which is to effectively diagnosis and treat 100% of estimated malaria cases [29]. Targeting those foci with higher expected prevalence within forested areas will become particularly important for Bangladesh.

## Conclusions

This study indicates that prevalence in Rajasthali remains high. Through identification of high-risk malaria zones one may generate new hypotheses regarding the spatial distribution of malaria. It can also be used as advocacy for channeling more funds to conduct operational research in high-risk areas. Targeting of interventions at such fine spatial scales will be helpful for the malaria control programme. Given the social and socio-economic risk factors are identified, more consideration could be given to methods of improving equity of access to interventions among whole communities in the region.

## Authors' contributions

Conceived the study design: UH, RJSM. Data preparation: UH. Data analysis: UH, DM. Prepared the manuscript: UH, GG. Conception, overall scientific management, interpretation of result, and critically reviewed the final report: KK, RH, RJSM, WP, GG. All authors approved the final version of the manuscript. Principal investigator of the project: UH.
